# Volatile uptake, transport, perception, and signaling shape a plant’s nose

**DOI:** 10.1042/EBC20210092

**Published:** 2022-09-30

**Authors:** Lei Wang, Matthias Erb

**Affiliations:** Institute of Plant Sciences, University of Bern, Altenbergrain 21, 3013 Bern, Switzerland

**Keywords:** defense signaling, MAPK, plant volatiles, transport, uptake, volatile perception

## Abstract

Herbivore-induced plant volatiles regulate defenses in undamaged neighboring plants. Understanding the mechanisms by which plant volatiles are taken up, perceived, and translated into canonical defense signaling pathways is an important frontier of knowledge. Volatiles can enter plants through stomata and the cuticle. They are likely perceived by membrane-associated receptors as well as intracellular receptors. The latter likely involves metabolization and transport across cell membranes by volatile transporters. Translation of volatiles into defense priming and induction typically involves mitogen-activated protein kinases (MAPKs), WRKY transcription factors, and jasmonates. We propose that the broad range of molecular processes involved in volatile signaling will likely result in substantial spatiotemporal and ontogenetic variation in plant responsiveness to volatiles, with important consequences for plant–environment interactions.

## Introduction

Plants produce and emit volatile organic compounds to mediate interactions with other organisms [1]. Upon insect herbivory, plants emit a complex blend of herbivore-induced plant volatiles [2,3]. These plant volatiles typically include green leave volatiles (GLVs), terpenoids, and phenylpropanoid/benzenoid volatiles. Based on the plant species and the herbivores that trigger the emission, the volatile blends differ in composition, quantity, and timing. Herbivore-induced plant volatiles play critical roles shaping the interactions between plant–insect herbivores, directly or indirectly. They function directly by triggering or mediating defense response in plants, or by acting as toxins or repellents against herbivores. They also function indirectly by attracting the natural enemies of insect herbivores [2–4].

A particularly interesting function of herbivore-induced plant volatiles is their ability to mediate defense in the systemic undamaged tissues or neighboring undamaged plants. [5–7]. Many volatiles have been shown capable of mediating interactions between plants (see a recent review for a comprehensive summary) [8]. Other than the phytohormone precursors MeJA and MeSA and the volatile hormone ethylene, GLVs are likely the most conserved volatile signals mediating plant–plant interactions. They trigger an array of defense responses in many plants, such as *Arabidopsis*, tomato, lima bean, and maize [9]. GLVs treatment in maize triggers the expression of defense-related genes and biosynthesis of defense-related metabolites, including many plant volatiles [10,11]. The emission of these volatiles further contributes to indirect defense [2]. In sweet potato, the homoterpene (E)-4,8-dimethyl-1,3,7-nonatriene (DMNT) can induce expression of proteinase inhibitor genes and increase herbivore resistance [12]. Volatiles such as indole and linalool typically do not induce defense directly, but they can prime several plants for stronger defense upon insect herbivory [11,13,14].

Recent years have seen substantial progress in understanding the biosynthesis, emission, and ecological function of plant volatiles. The perception of plant volatiles is now also being unraveled [1–3,15]. To fully understand how volatiles mediate plant–plant interactions, it is important to address how volatiles enter plant tissues and get recognized thereafter. A few recent reviews have summarized the latest discoveries in volatile biosynthesis, emission, and bioactivity [1,8,16]. Here, we discuss the possible paths that volatiles may take to enter leaf tissues. We review the current evidence regarding the role of receptors/receptor complexes in volatile perception, with a focus on herbivore-induced plant volatiles. We further summarize the downstream signaling events that are triggered by herbivore-induced plant volatiles, and explore the hypothesis that spatiotemporal variation in volatile uptake, transport, perception, and signaling shape a plant’s ‘nose’, i.e. the tissues that are involved in perceiving volatiles as environmental cues. Pathogen-induced volatiles are covered in a separate paper in the same issue by Vlot and colleagues [17].

## Entry of volatiles into plant leaves

**Figure 1:**
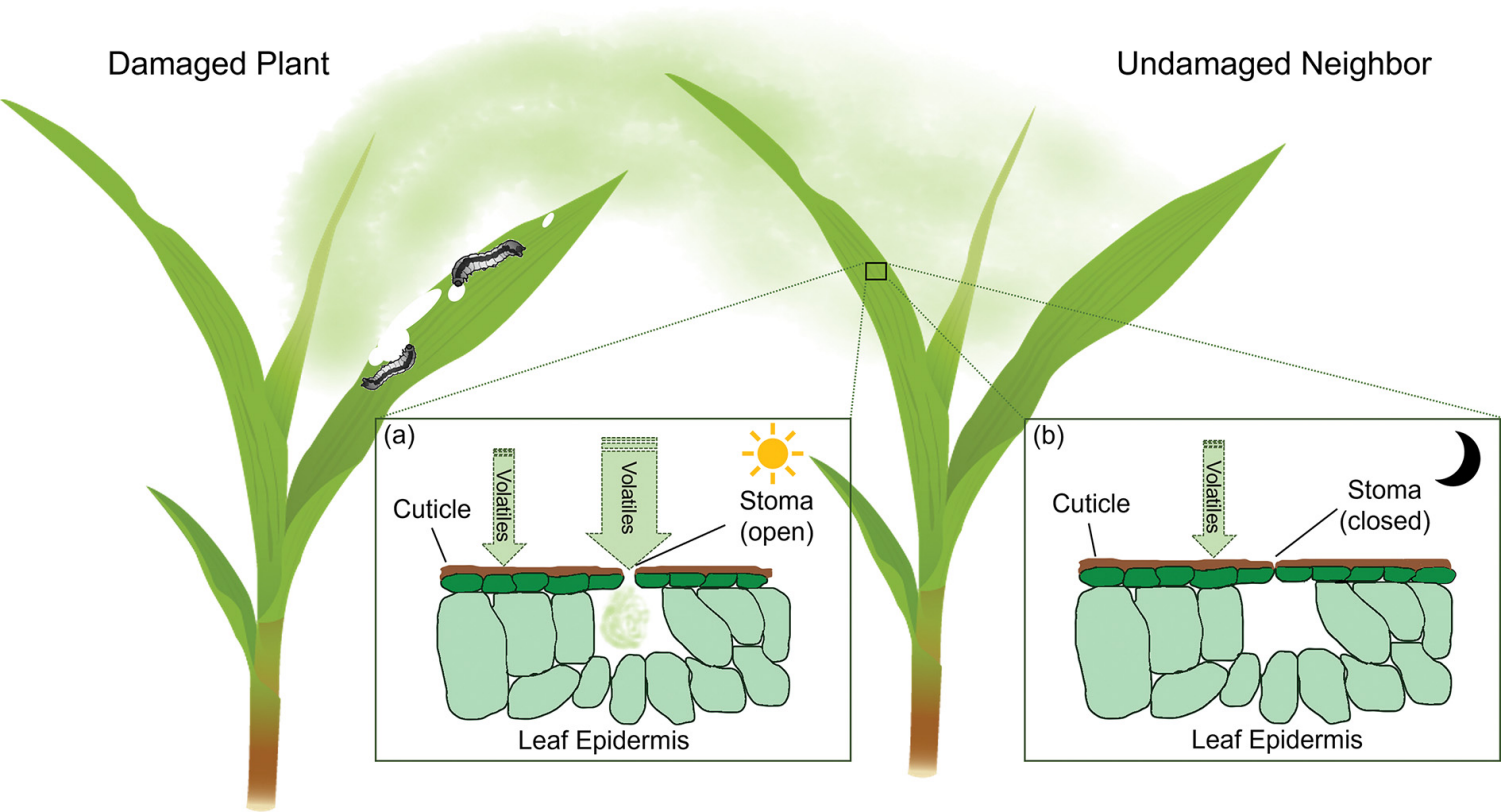
Schematic view of possible volatile entry routes into leaf tissues Herbivore-induced volatiles emitted from damaged plants may enter the plant tissue of undamaged neighbors through stomata and/or diffuse through the cuticle. (**a**) During daytime, stomata may be the main entry sites due to low resistance. (**b**) At night, when the stomata are closed, diffusion through the cuticle may become more important.

To trigger defense response in the cells, volatiles need to access the plasma membrane or intracellular compartments. Volatiles may enter the leaves either through stomata or pass through the cuticular wax layer (Figure 1).

### Entry through stomata

Stomata are the breathing pores of plant leaves, balancing photosynthetic carbon dioxide uptake and evaporative water loss [18]. These natural openings provide entry low resistance points for environmental agents such as microbes [19]. Recent studies show that stomata also shape plant–insect interactions by controlling volatile emission [20–22]. In maize, stomatal closure, induced by darkness or abscisic acid (ABA) treatment, constrains the emission of elicitor-triggered sesquiterpenes. Maize plants induced by elicitors in the dark show a burst of sesquiterpenes emission when light is switched on, indicating stomata as the gate for these volatiles to be released into the atmosphere [20]. Similarly, tomato and soybean leaves emit less volatiles when the stomata are partially closed by glucose oxidase, a salivary protein from the caterpillar *Helicoverpa zea* [21].

Given the considerations above, it is reasonable to assume that stomata also serve as low-resistance entry points for volatiles. Volatiles from attacked plants may thus enter the leaves of neighboring plants through these openings. If stomata are the main entry path for plant volatiles, several aspects need to be considered. First, as plants close their stomata at night [23], this would imply that plants are not able to perceive volatiles during nighttime, despite the fact that herbivore-induced plant volatiles such as GLVs are released as danger cues at night [24]. Another important aspect to consider is the developmental stage of stomata in leaves. In grasses, for instance, the stomatal complex is under differentiation and formation in the developmental zone in the young leaves [18]. Thus, if stomata are important for volatile uptake, the ability of young leaves to respond to volatiles will differ from that of the old leaves, where stomata are fully developed. Experiments that investigate diurnal and developmental variation in volatile perception could thus provide first indications on the potential role of stomata in volatile perception.

### Entry through the cuticle

The plant cuticle is the final barrier for volatiles to be released into atmosphere from nonvegetative organs [16]. In petunia flowers, the cuticle acts both as a resistance barrier and a sink for VOCs. Its thickness thus affects the dynamics of volatile emission [25]. This effect also depends on the physiochemical properties of VOCs, with volatiles having lower ambient vapor pressure facing higher resistance [16,26]. Several plants use their leaves to adsorb/take up volatiles from neighboring plants for enhanced herbivore resistance [27]. A recent study shows that plant leaf cuticular waxes can sequester exogenous volatiles [28]. Thus, it can be hypothesized that plant volatiles may pass the leave cuticle and diffuse across the more permeable cell wall to reach the plasma membrane, effectively bypassing stomata.

Plant cuticular waxes comprise mainly very long-chain fatty acids and their derivatives. Both the wax composition and structure change greatly during leaf development. In wheat, leaf surface wax keeps accumulating until the leaf blade finishes expanding. Meanwhile, the carbon chain length of the wax constituents increases drastically, and the wax crystals form different structure [29]. These changes likely cause different volatile permeability in young leaves and old leaves. Different physiochemical properties of volatiles will further increase the variance. So far, responses in volatile perception are either analyzed on the whole plant level or on a specific leaf. Experiments with cuticle mutants will help to uncover the role of cuticle as entry sites for volatiles relative to stomata.

## Volatile perception at the cell membrane

Once accessing the plasma membrane, VOCs may be perceived by receptor/receptor complexes to trigger cellular response or taken up for further metabolic processing. Finding plant volatile receptors has been a long-standing question, but a breakthrough has yet to be achieved [3,15]. Ample progress has been made on identifying receptors for microbe-associated molecular patterns (MAMPs), herbivore-associated molecular patterns (HAMPs), and damage-associated patterns (DAMPs) [30,31]. These discoveries may inspire the discovery of a plant volatile receptors.

GLVs, C6 aldehydes, alcohols, and esters are enzymatically generated from membrane lipids upon disruption of membrane integrity in leaves, upon mechanical wounding or insect feeding [9]. Based on the plant origin and their ability to induce defense, these fatty acid-derived molecules can also be termed as DAMPs [32]. In *Arabidopsis*, the lectin receptor kinase LORE recognizes bacterial medium-chain 3-hydroxy fatty acid as a MAMP to trigger immunity [33]. Another *Arabidopsis* lectin receptor kinase LecRK-I.8 is critical for the defense triggered by phosphatidylcholines primarily with C16- to C18-fatty acyl chains [34]. The maize ZmFACS protein, a leucine-rich repeat (LRR) receptor kinase, mediates defense triggered by the fatty acid–amino acid conjugates (FACs) [35]. GLVs, as fatty acid derivatives, share certain biochemical properties with the molecular patterns mentioned above. Thus, they may be perceived by plasma membrane-localized receptor kinases or receptor proteins as well. Typically, these receptors need to form a protein complex with coreceptors to achieve full immune responses. These coreceptors are often from the somatic embryogenesis receptor kinase (SERKs) family [31]. Thus, screening lectin/LRR receptor kinase/receptor protein mutants and SERKs mutants for abolished or reduced GLVs response may help identifying components of the hypothesized GLVs receptor complex.

## Volatile uptake into cells

MAMPs and DAMPs receptors are plasma membrane-localized receptors [31]. Perception of danger-related molecules can also happen inside of plant cells, such as the perception of effectors by nucleotide-binding domain leucine-rich repeat-containing (NLR) proteins [36]. It is possible that perception of some VOCs happens intracellularly. Additionally, plants take up volatiles directly for further metabolic processing [27]. In either case, these volatiles need to pass through the plasma membrane. Direct diffusion may happen for membrane lipid derived volatiles such as GLVs. This is unlikely for most other volatiles [16]. In petunia, transport of VOCs across the plasma membrane relies on an adenosine triphosphate-binding cassette (ABC) transporter [37]. Similar volatile transporters may mediate the channeling of volatiles from the extracellular into the intracellular space. Transporters or ion channels may act as receptors as well. For example, the *Arabidopsis* anion channel SLAC1 plays an important role in sensing CO_2_/bicarbonate in the guard cells [38]. Within the cells, volatiles may bind specific proteins to initiate cellular responses. Recently, the transcription regulators TOPLESS-like proteins (TPLs) were found to bind the sesquiterpene caryophyllene [39]. However, it is unclear whether TPLs alone are sufficient to act as receptors to transduce caryophyllene-triggered responses. So far, hypothetical plant odorant-binding proteins (OBPs) have also been proposed to act as plant volatile receptors, based on their similarity with animal OBPs. A recent study used *in silico* molecular docking to prove plant OBPs can bind monoterpenes [40]. The specificity and *in vivo* activity of OBPs remains to be uncovered.

## Translation into defense signaling

**Figure 2:**
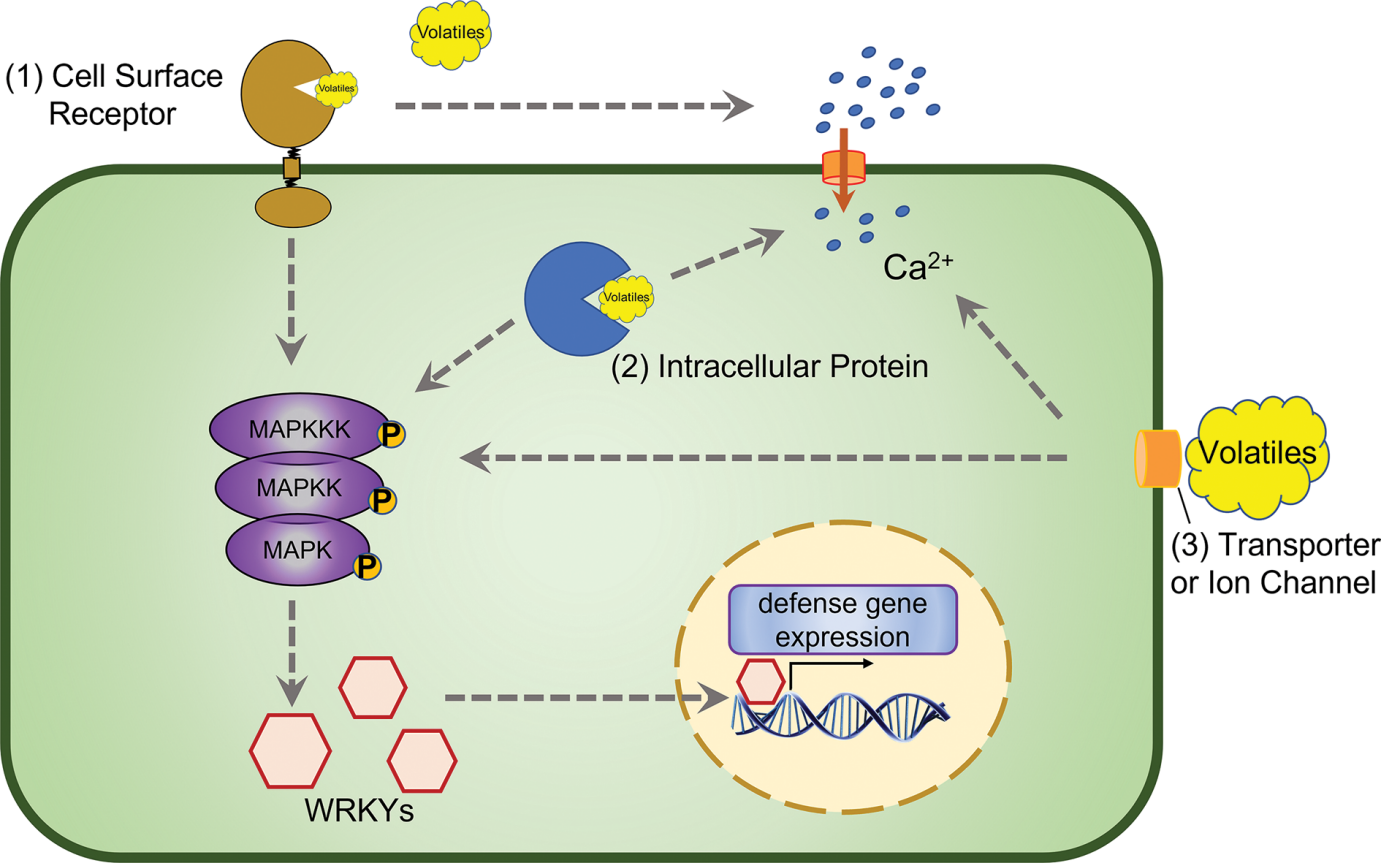
Schematic representation of plant volatile perception and signaling Plant volatiles are likely perceived in three different ways. The respective responsible proteins are: (1) cell surface receptors, (2) intracellular proteins, and (3) volatile transporters or ion channels. Volatile perception leads to a cascade of defense responses, including calcium influx, MAPK activation, and WRKY transcription factor-regulated expression of defense genes. Dashed arrows indicate unclear molecular mechanisms of the signaling cascade.

Volatiles are well established to trigger defense pathways that are typically associated with MAMPs and DAMPs [8,30] (Figure 2).

Upon exposure to GLVs, *Arabidopsis* and tomato plants show a cytosolic calcium influx. In *Arabidopsis*, this transient influx happens rapidly upon GLVs exposure and peaks at 5–10 min [41]. The calcium influx kinetic is unclear in tomato likely due to the lack of a calcium reporter line [42]. Additionally, ocimene, myrcene, pinene, and DMNT can also trigger transient cytosolic calcium influx in *Arabidopsis*, similar as the one triggered by GLVs [41]. Calcium influx is a typical early response, following the perception of MAMPs and DAMPs. NLR activation after effector recognition also leads to calcium influx, *albeit* with different dynamics [36]. Collectively, these studies indicate calcium influx is a conserved immune response upon danger perception, including volatiles.

Activation of mitogen-activated protein kinases (MAPKs) is another classical early immune response upon pattern recognition [43]. Similarly, GLV exposure leads to rapid activation of MAPKs in the grass *Lolium temulentum* [44]. The sesquiterpene (E)-Nerolidol increases both the transcript and protein of MAPK in tea plants [45]. Indole exposure does not activate MAPKs directly but increases MAPK gene expression and activation following simulated herbivory in rice. Knocking down *MPK3* and *MPK6* leads to abolished or greatly reduced defense priming effect by indole [46]. MAPK cascades are also points of convergence between different signaling pathways [47]. This role may explain the phenomenon that dual exposure of the GLV (Z)-3-hexenyl-acetate (HAC) and indole in maize generates stronger defense than HAC exposure alone [11].

Other commonly reported volatile responses include the increased expression of genes-encoding transcription factors, defense hormone biosynthesis enzymes, and defense metabolite biosynthesis enzymes [8]. Members of the WRKY transcription factor family are often induced by various biotic and abiotic stress and regulate hormone biosynthesis in turn [48,49]. In *Arabidopsis*, the GLV (E)-2-hexenal induces the expression of several *WRKY* genes, including *AtWRKY6*, *AtWRKY40*, and *AtWRKY53*. Knocking out these genes leads to increased expression of (E)-2-hexenal-specific responsive genes, indicating that these transcription factors as negative regulators of GLVs signaling in *Arabidopsis* [48]. The GLV (Z)-3-hexenol increases *ZmWRKY12* transcripts in maize, but the importance of this phenomenon is unclear [50]. Similarly, *CsWRKY3* expression is up-regulated by (E)-Nerolidol but its role in (E)-Nerolidol signaling is unknown [45].

Plant VOCs may also suppress defense in some cases [14,51]. Whitefly-infested tomato plants emit a unique blend of volatiles to suppress JA-dependent defense but prime SA-dependent defense. The dual role in defense is triggered by two terpenes: β-myrcene or β-caryophyllene. On the contrary, linalool, a monoterpene elicited by the *Spodoptera exigua* caterpillar attacked tomato plants, primes the expression of two JA-pathway proteinase inhibitor genes, *PI-I and PI-II* [14]. The molecular mechanisms behind these differences are unknown. Future work on how volatile-triggered defense is differentially regulated will greatly help to uncover novel volatile signaling components and pathways.

## Spatiotemporal patterns of volatile perception

Since the discovery of ‘talking trees’, plant volatile-mediated plant–plant interactions have fascinated many scientists and let to substantial research efforts [5]. We now have a detailed understanding of plant volatile biosynthesis and transport [1,16]. Based on this knowledge, we can infer that plant volatile perception likely involves a number of physical structures such as stomata and cuticles as well as molecular elements such as transporters, receptors, and signal integration proteins. Given the substantial variation in the expression of these elements in different plant parts and developmental stages, we predict that plant volatile perception will not be uniform, but will show significant variation within a given plant. Certain leaves are likely to be much more sensitive to others, and could thus be viewed as a plants ‘nose’. Understanding these patterns and linking them to our increased understanding of the mechanisms of plant volatile perception will be important to unravel the ecological dynamics that are elicited by volatiles and to exploit plant volatiles as crop-reprogramming signals. As comprehensively summarized in a recent review, the potential applications of plant volatiles for sustainable agricultural practices will include breeding crops with enhanced volatile emissions and inducing volatile release in a targeted manner [2]. A good understanding of the mechanisms and spatiotemporal variation in volatile perception will also facilitate the selection and breeding of plants that are sensitive to plant volatiles in the right place at the right time. Such work will help to unlock the potential of plant volatiles as crop-reprogramming agents.

## Summary

- Plant volatiles may enter the inner space of plant leaves through stomata or cuticle. The relative importance of these entry sites is likely to vary with development and environmental conditions.
- Plant volatile perception is likely mediated by cell surface receptors, plasma membrane-localized transporters or ion channels, and intracellular proteins.
- Plant volatiles regulate canonical defense signaling pathways, with MAPKs and WRKY transcription factors playing important roles as signal integration hubs.
- Spatiotemporal variation in volatile uptake and perception elements will likely determine where and when plants respond to volatiles.

## Abbreviations

ABA: abscisic acid
ABC: adenosine triphosphate-binding cassette
DAMP: damage-associated molecular pattern
DMNT: (E)-4,8-dimethyl-1,3,7-nonatriene
FAC: fatty acid–amino acid conjugate
GLV: green leave volatile
HAC: (Z)-3-hexenyl-acetate
HAMP: herbivore-associated molecular pattern
JA: jasmonic acid
LRR: leucine-rich repeat
MAMP: microbe-associated molecular pattern
MAPK: mitogen-activated protein kinase
NLR: nucleotide-binding, leucine-rich repeat
OBP: odorant-binding protein
SA: salicylic acid
SERK: somatic embryogenesis receptor kinase
TPL: TOPLESS-like proteins
VOC: volatile organic compound
